# Perceived Barriers, Facilitators and Benefits for Regular Physical Activity and Exercise in Patients with Rheumatoid Arthritis: A Review of the Literature

**DOI:** 10.1007/s40279-015-0363-2

**Published:** 2015-07-29

**Authors:** Jet J. C. S. Veldhuijzen van Zanten, Peter C. Rouse, Elizabeth D. Hale, Nikos Ntoumanis, George S. Metsios, Joan L. Duda, George D. Kitas

**Affiliations:** School of Sport, Exercise and Rehabilitation Sciences, University of Birmingham, Birmingham, B15 2TT UK; Department of Rheumatology, Dudley Group NHS Foundation Trust, Dudley, UK; Department of Health, University of Bath, Bath, UK; School of Psychology and Speech Pathology, Curtin University, Perth, WA Australia; School of Sport, Performing Arts and Leisure, University of Wolverhampton, Wolverhampton, UK

## Abstract

Rheumatoid arthritis (RA) is an autoimmune disease, which not only affects the joints but can also impact on general well-being and risk for cardiovascular disease. Regular physical activity and exercise in patients with RA have numerous health benefits. Nevertheless, the majority of patients with RA are physically inactive. This indicates that people with RA might experience additional or more severe barriers to physical activity or exercise than the general population. This narrative review provides an overview of perceived barriers, benefits and facilitators of physical activity and exercise in RA. Databases were searched for articles published until September 2014 using the terms ‘rheumatoid arthritis’, ‘physical activity’, ‘exercise’, ‘barriers’, ‘facilitators’, ‘benefits’, ‘motivation’, ‘motivators’ and ‘enablers’. Similarities were found between disease-specific barriers and benefits of physical activity and exercise, e.g. pain and fatigue are frequently mentioned as barriers, but reductions in pain and fatigue are perceived benefits of physical activity and exercise. Even though exercise does not influence the existence of barriers, physically active patients appear to be more capable of overcoming them. Therefore, exercise programmes should enhance self-efficacy for exercise in order to achieve long-term physical activity and exercise behaviour. Encouragement from health professionals and friends/family are facilitators for physical activity and exercise. There is a need for interventions that support RA patients in overcoming barriers to physical activity and exercise and help sustain this important health behaviour.

## Key Points

**Table:** 

Patients with rheumatoid arthritis (RA) who exercise regularly and those who do not, report similar barriers to physical activity and exercise but different coping strategies.
Support from healthcare providers and family/friends is an important facilitator for physical activity in RA.
Knowledge about appropriate exercise programmes is lacking in RA patients and healthcare providers.

## Introduction

Rheumatoid arthritis (RA) is an autoimmune disease affecting approximately 1 % of the general population. It causes joint pain, stiffness and swelling, and can lead to permanent structural joint damage and deformity, while it may also be associated with systemic complications from major organ systems including the heart, lungs and blood vessels [1]. RA impacts on the patient’s life in several ways: apart from the physical limitations, employment roles and psychological well-being may also be negatively affected. For example, RA patients tend to experience high levels of fatigue [2, 3], many are unable to continue gainful employment [4] and many have depression [5, 6]. In addition, patients with RA have an increased risk for cardiovascular disease (CVD) [7–9]. This could be attributed to the increased prevalence of classical CVD risk factors, such as hypertension [10], dyslipidaemia [11] and obesity [12], and to direct vascular effects of systemic inflammation [13]. The disease is treated with a variety of medications that aim to improve symptoms and stop or decelerate structural joint damage. However, given that physical activity and exercise have been shown to successfully improve general well-being [14] and reduce the risk for CVD in the general population [15], it is not surprising that engagement in regular physical activity and exercise has been recommended as part of the overall multidisciplinary management of RA patients [16, 17].

There is ample evidence that physical activity and exercise in patients with RA has numerous health benefits, such as improving joint health, physical function, mobility and psychological well-being, as well as reducing rheumatoid cachexia and fatigue without aggravating symptoms or inducing further joint damage (for reviews, see Cooney et al. [16], Summers et al. [17], Metsios et al. [18, 19] and Cramp et al. [20]). More recently, individualised exercise regimens have been shown to reduce cardiovascular risk and vascular function in RA [21, 22]. Even though RA patients report awareness of the positive effects of physical activity and exercise on their general health [23–27], systematic reviews have revealed that physical activity levels are lower in RA than healthy control participants [28]. Moreover, the physical activity levels are lower than physical activity recommendations [28], with 71 % of RA patients not participating in regular physical activity [29]. A recent randomised controlled trial showed that a cognitive behavioural patient education programme was successful in increasing the intentions to become physically active, but, unfortunately, physical activity behaviour was not changed as a result of this intervention [30]. Together, these reports could indicate that compared to the average person, RA patients may face more severe or additional barriers that contribute to reducing their levels of physical activity to well below those observed in the general population [28]. Identifying and understanding these barriers is important in order to facilitate the development of effective programmes and interventions that result in sustainable physical activity and subsequent health benefits in people with RA. This narrative review aims to provide an overview of the perceived barriers, facilitators and benefits of physical activity as well as exercise and their impact on physical activity behaviour in patients with RA.

## Literature Search Methodology

Databases (MEDLINE, Web of Science) were searched to identify articles published until September 2014 regarding RA and physical activity. Specific terms that were used were ‘rheumatoid arthritis’ AND ‘physical activity’/‘exercise’ AND ‘barriers’/‘facilitators’/‘benefits’/‘motivation’/‘motivators’/‘enablers’. Full articles were retrieved for assessment if the abstract fulfilled the following criteria: (a) it was indicated that barriers, benefits or facilitators for physical activity or exercise were assessed; and (b) the sample(s) included RA patients. Studies that incorporated other types of arthritis were also included, as long as RA patients were in the sample. Additional published papers were found on the basis of the reference lists. Conference proceedings were not included in this review. Literature search and data extraction was carried out by JVvZ. All procedures were in line with published guidelines for writing a narrative review [31].

All studies found were cross-sectional, and have employed a variety of both qualitative and quantitative methods to explore the barriers, facilitators or benefits of physical activity. To give a comprehensive overview of the available literature, this review reports data from both methodologies. Qualitative research methods include interviews and focus groups, and allow a wider interpretation and in-depth examination of barriers, facilitators and benefits of physical activity and exercise. Therefore, the data generated from qualitative studies provide great depth but are usually limited to small numbers of participants. In quantitative approaches, which typically involve a larger number of participants, the patients are provided with structured questionnaires containing pre-identified arthritis-specific as well as generic barriers, facilitators or benefits of physical activity and exercise. The patients are then asked to identify the presence and, in some cases, rate the impact of these perceived barriers and benefits on physical activity. This quantitative approach establishes the strength or relevance of existing barriers, facilitators and benefits as they relate to engagement in physical activity and exercise. We first report the perceived barriers, facilitators and benefits of physical activity and exercise, followed by a description of their associations with physical activity behaviour.

## Findings

**Fig. 1:**
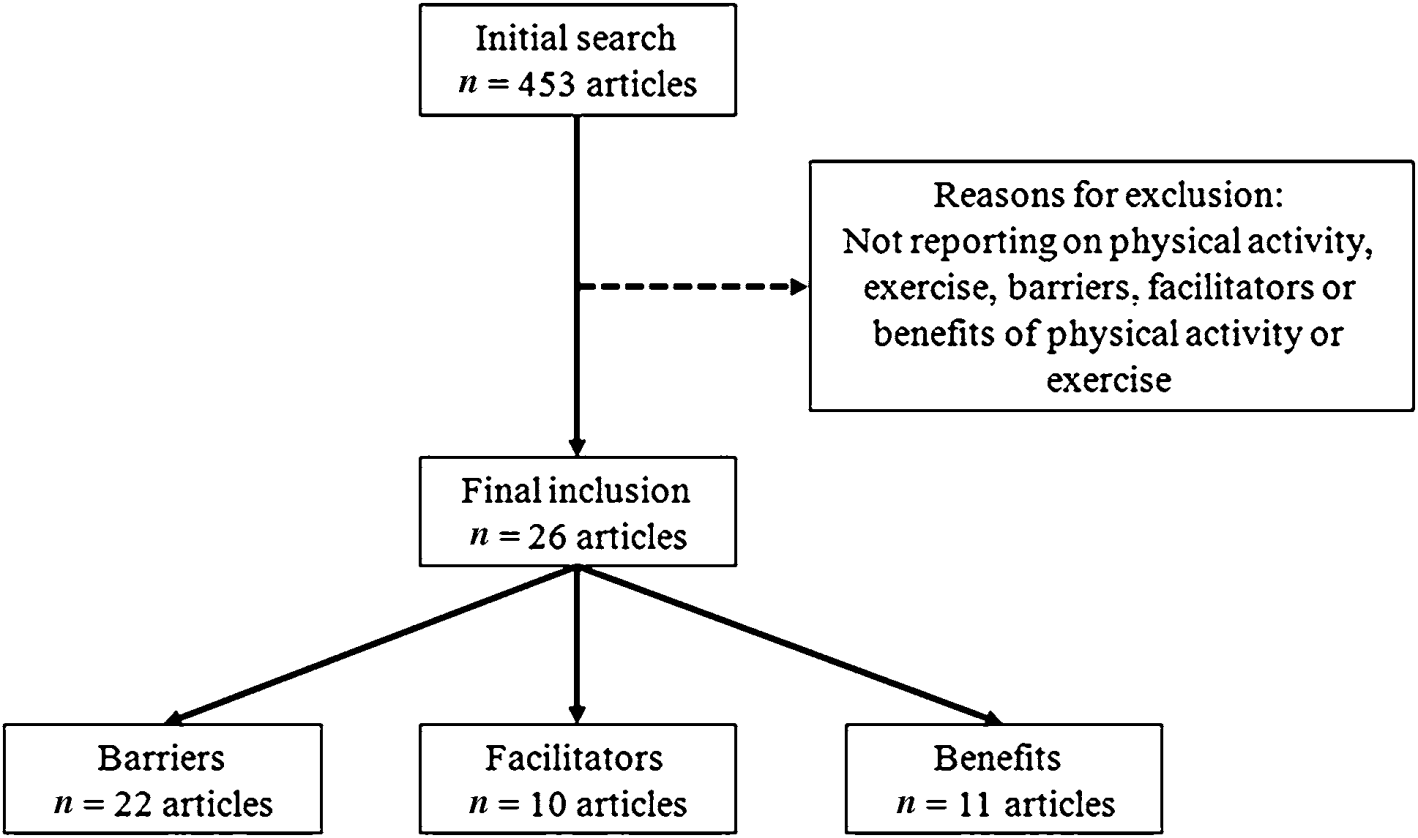
Flow chart of the literature search

In total 453 articles were found, of which 26 fulfilled the inclusion criteria of the review. The most common reasons for exclusion were not reporting on physical activity, exercise barriers, facilitators or benefits (see Fig. 1). An overview of the included studies relating to perceived barriers, facilitators and benefits of physical activity is presented in Tables 1, 2 and 3, respectively. The data presented in these tables reveal that studies vary with regard to the inclusion criteria of RA patients. For example, whereas some studies only report data from physician-diagnosed RA, others include those with a self-reported diagnosis of RA, or include patients with several types of arthritis, including RA. Additional analyses are reported for the ten studies that limited their recruitment to RA patients with a confirmed physician diagnosis of RA. Differences in sample size and assessment methods (quantitative or qualitative) are also evident from the tables. It is worth noting that, with one exception [32], all studies were conducted in Western European countries or the USA.

**Table 1:** Overview of studies that reported perceived barriers to physical activity and exercise in patients with rheumatoid arthritis

Study	Participants (*n*)	Assessment	Findings—RA specific	Findings—generic
Quantitative studies				
Stenstrom et al. [33]	79 RA—physician diagnosis (ACR)	Questionnaires	Pain	Time
Neuberger et al. [34]	100 (63 diagnosed RA, 37 OA—physician diagnosis)	Questionnaires	Inaccessibility of exercise facilities, no encouragement, exercise too tiring	Inconvenient schedule, time, effort
Jensen and Lorish [35]	305 rheumatology clinics (RA, OA, back pain—self-diagnosis)	Questionnaires	Lack of desired results, made more tired, joints felt worse	Got out of habit, boring/not fun
Iversen et al. [36]	140 RA—physician diagnosis (ACR)	Questionnaires	Pain	Time, boring
Kang et al. [32]	72 arthritis (12 RA)—physician diagnosis	Questionnaires	No convenient facility/place	Location of pool
Van den Berg et al. [37]	252 RA—physician diagnosis (ACR): 80 % active, 20 % inactive	Questionnaires	Lack of energy, pain, fear of damaging joints (no difference between physically active and inactive patients)	Lack of motivation
Bajwa and Rogers [38]	223 arthritis—self-reported diagnosis of arthritis	Interviews	Bad health, pain	
Martin et al. [50]	1292 arthritis—self-reported diagnosis of arthritis	Interviews	Ill or otherwise physically unable	
Hutton et al. [39]	1106 arthritis—self-reported diagnosis of arthritis 1106 age-, sex- and ethnicity-matched controls	Questionnaires	Arthritis/other health problems, lack of energy/too tired	
Gyurcsik et al. [40]	80 arthritis—physician diagnosis	Questionnaires	Fatigue, pain	
Brittain et al. [41]	248 arthritis—self-reported diagnosis	Questionnaires (online)	Pain, arthritis limits body capability, stiffness	Temperature, too tired after work, time
Law et al. [26]	247 RA—self-reported diagnosis	Questionnaires (online)	Worry about causing harm, joint pain	
Henchoz et al. [27]	89 RA—physician diagnosis (ACR) (34 % no regular exercise, 45 % regular exercise)	Questionnaires	Pain, stiffness, tired, arthritis-related limitations	
Qualitative studies				
Hammond [42]	41 RA—physician diagnosis (ACR)	Interviews	Pain	Time/motivation, getting sufficient exercise already in job/household
Kamwendo et al. [43]	10 RA—physician diagnosis	Interviews	Tiredness, pain, fatigue, fear of pain, external barriers, lack of instructions	Time, environmental barriers (e.g. weather)
Lambert et al. [24]	12 arthritis—physician diagnosis	Focus groups	Uncertainty about safe exercise and injury prevention	
Schoster et al. [44]	36 arthritis completers of exercise program, 15 arthritis non-completers of exercise program—self-reported diagnosis	Interviews	Personal illness (non-completers arthritis related, completers general illness), class not challenging (non-completers)	
Wilcox et al. [45]	26 arthritis exercisers (14 RA), 32 arthritis non-exercisers (8 RA)—self-reported diagnosis	Focus groups	Pain, fatigue, mobility, co-morbid conditions, attitudes and beliefs, fear of pain, perceived negative outcomes, lack of support, no one to exercise with, lack of programmes/facilities	Competing roles/responsibilities, environmental conditions, cost, transportation
Der Ananian et al. [46]	15 arthritis non exercisers (4 RA), 15 arthritis insufficiently active (3 RA), 16 arthritis exercisers (6 RA)—self-reported diagnosis	Focus groups	Pain, mobility, co-morbidities, fatigue, attitudes and beliefs (e.g. lack of exercise knowledge, reducing pain/symptoms), perceived negative outcomes, insufficient advice from healthcare providers, lack of exercise programmes	Competing roles/responsibilities, attitudes and beliefs (e.g. laziness, lack of enjoyment/time), weather
Martin et al. [50]	19 arthritis—self-reported diagnosis	Focus group and interviews	Personal health, chronic illness	Cost
Swardh et al. [47]	18 RA—physician diagnosis	Interviews	Pain, fatigue	Time, cost and cold climate
Law et al. [48]	18 RA—physician diagnosis	Focus groups	Lack of knowledge of health professional and patient, joint and muscle pain, worry about causing harm to joint, fatigue	Lack of enjoyment, motivation and confidence
Kaptein et al. [49]	40 arthritis—self-reported diagnosis	Focus groups	Lack of knowledge about exercise, pain, unpredictable nature of arthritis, fatigue	Competing roles

*ACR* American College of Rheumatology, *OA* osteoarthritis, *RA* rheumatoid arthritis

**Table 2:** Overview of studies that reported perceived facilitators of physical activity and exercise in patients with rheumatoid arthritis

Study	Participants (*n)*	Assessment	Findings—RA specific	Findings—generic
Quantitative studies				
Stenstrom et al. [59]	95 rheumatic condition (35 inflammatory arthritis)—self-reported diagnosis	Questionnaires	Psychological factors most important, then physiological factors and social factors Psychological motivation and physical motivation equally important, then social motivation	
Hutton et al. [39]	1106 arthritis—self-reported diagnosis 1106 age-, sex- and ethnicity-matched controls	Questionnaires	Want to take responsibility	Childcare, time (allowance by employer), companion
Qualitative studies				
Kamwendo et al. [43]	10 RA—physician diagnosis	Interviews	Strength and aerobic capacity, self-efficacy, support from healthcare providers and friends/family, improvement in stiffness, fear of getting worse, intimidation when confronted with worse RA	Happiness, social benefits, personal satisfaction
Schoster et al. [44]	36 arthritis completers of exercise programme, 15 arthritis non-completers of exercise programme—self-reported diagnosis	Interviews	Instructor support, self-efficacy, exercise information	Class social support
Wilcox et al. [45]	26 arthritis exercisers (14 RA), 32 arthritis non-exercisers (8 RA)—self-reported diagnosis	Focus groups	Encouragement of significant other, programmes/knowledgeable instructors	Internal motivation, social interaction, exercise buddy, low-cost programmes
Der Ananian et al. [46]	15 arthritis non-exercisers (4 RA), 15 arthritis insufficiently active (3 RA), 16 arthritis exercisers (6 RA)—self-reported diagnosis	Focus groups	Social support from significant other/people with arthritis, healthcare provider advice, access to exercise programmes with knowledgeable instructors	Making exercise a priority, self-motivation
Swardh et al. [47]	18 RA—physician diagnosis	Interviews	Feeling of safety, support/guidance, encouragement of instructor, feeling of autonomy	
Law et al. [48]	18 RA—physician diagnosis	Focus groups	Assistance from instructors	Social interaction, low cost, easy access, weight reduction
Kaptein et al. [49]	40 arthritis—self-reported diagnosis	Focus groups	Social support	
Loeppenthin et al. [58]	16 RA—self-reported diagnosis	Interviews	Support/motivation from others (including healthcare professionals)	

*RA* rheumatoid arthritis

**Table 3:** Overview of studies that reported perceived benefits of physical activity and exercise in patients with rheumatoid arthritis

Study	Participants (*n*)	Assessment	Findings—RA specific	Findings—generic
Quantitative studies				
Hutton et al. [39]	1106 arthritis—self-reported diagnosis 1106 age-, sex- and ethnicity-matched controls	Questionnaires	Good for health	Enjoyment, taking responsibility, role model for children
Iversen et al. [25]	113 RA—physician diagnosis (ACR)	Questionnaires	Pain relief	
Jensen and Lorish [35]	305 rheumatology clinics (RA, OA, back pain)—self-diagnosis	Questionnaires	Make joints feel better, able to do other tasks more easily, feel more in control, showing family/friends that I can do them	Feel better overall, pleasing person who prescribed exercise
Law et al. [26]	247 RA—self-reported diagnosis	Questionnaire (online)	Helpful	
Henchoz et al. [27]	89 RA—physician diagnosis (ACR) (34 % no regular exercise, 45 % regular exercise)	Questionnaire	Physical benefits (e.g. decreases stiffness), psychological benefits (e.g. better endure pain), functional benefits (e.g. functional ability and independence)	Physical benefits (e.g. lose weight), psychological benefits (e.g. pleasure), social benefits (e.g. spend time with friends and family)
Qualitative studies				
Eurenius et al. [62]	16 RA—physician diagnosis (ACR)	Interviews	Preventing decline, maintaining physical capacity	Increase confidence
Lambert et al. [24]	12 arthritis—physician diagnosis	Focus groups	Exercise important factor in treatment, helpful to get away from pain	
Wilcox et al. [45]	26 arthritis exercisers (14 RA), 32 arthritis non-exercisers (8 RA)—self-reported diagnosis	Focus groups	Symptom management, mobility and function, strength and flexibility, independency, attitudes and beliefs	Weight loss, emotional benefits and enjoyment
Der Ananian et al. [46]	15 arthritis non-exercisers (4 RA), 15 arthritis insufficiently active (3 RA), 16 arthritis exercisers (6 RA)—self-reported diagnosis	Focus groups	Symptom management (more tolerable pain), improved mobility, independence	Feeling better, reducing stress
Kamwendo et al. [43]	10 RA—physician diagnosis	Interviews	Strength and aerobic capacity, prevention of stiffness, combat the fear of getting worse	Happiness, self-efficacy, social benefits
Loeppenthin et al. [58]	16 RA—self-reported diagnosis	Interviews	Joy, energy, preservation of bodily consciousness, manage illness, strength, functional ability, satisfaction, maintenance of independence and autonomy, sense of belonging	

*ACR* American College of Rheumatology, *OA* osteoarthritis, *RA* rheumatoid arthritis

### Perceived Barriers to Physical Activity and Exercise

Table 1 provides a detailed overview of the barriers that were identified by RA patients. Twenty-two studies were found, of which 12 used quantitative methodology [26, 27, 32–41], nine used qualitative methodology [24, 42–49] and one study reported on both quantitative and qualitative methods [50]. Both qualitative and quantitative studies reported several barriers that are not specifically related to their disease, such as lack of time [34, 36, 42, 43, 47, 49, 51, 52] and the cost of exercise [39, 45, 47, 50]. In total, 15 (seven quantitative studies, eight qualitative studies) of the 22 studies that described barriers to physical activity and exercise reported at least one non-arthritis-specific barrier, similar to the barriers frequently reported by non-clinical populations. Studies that only included patients with a confirmed diagnosis of RA reported that lack of time was the most consistently reported barrier in both quantitative and qualitative approaches [33, 36, 42, 43, 47], followed by lack of motivation [37, 42, 48]. Even though the impact of these generic barriers should not be ignored, given the aims of the current review, the present results centre on the disease-specific barriers that are reported in patients with RA.

It is evident from both the quantitative and qualitative studies that *pain* (15 of 22 studies) and *fatigue* (12 of 22 studies) were the most commonly identified arthritis-specific barriers to participating in regular physical activity and exercise [26, 27, 33–43, 45–49]. *Reduced mobility/functional ability* (4 of 22 studies) and *stiffness* (2 of 22 studies) were other disease-related barriers mentioned as impeding physical activity participation [27, 35, 39, 41, 45, 46]. In addition to these physical barriers, which are reported in both quantitative and qualitative studies, qualitative studies also mentioned that a lack of provision of exercise programmes geared towards patients with arthritis [43, 45, 46] and a lack of knowledge about exercise regimens that are appropriate for patients with arthritis [24, 48] were perceived to negatively influence physical activity behaviour. This lack of knowledge regarding appropriate physical activity and exercise for RA has been related to fear of aggravating the disease or damaging joints [37, 43, 45, 46, 48]. Patients also felt that healthcare providers are unclear about the suitability of different types of exercise programmes for RA [26, 46, 48]. Similar results were found when analysing the studies that included only patients with a confirmed RA diagnosis. Pain was identified as a barrier by all eight studies [27, 33, 36, 37, 42, 43, 47, 48] and fatigue by seven of eight studies [27, 33, 36, 37, 43, 47, 48], with two qualitative studies reporting lack of advice from the healthcare provider as a perceived barrier to physical activity [43] and exercise [48].

Both quantitative and qualitative approaches were used to compare patients who participate in regular physical activity or exercise regularly and those who do not. These studies revealed no difference in perceived arthritis-specific barriers between the two groups in most [32, 37, 45, 46, 53], but not all [27, 44], studies. However, although the RA-related barriers appeared to be similar, qualitative studies showed a striking difference between the coping strategies between exercisers and non-exercisers. Whereas exercisers knew how to adjust their physical activity when experiencing a flare in disease activity or a high level of fatigue, those with insufficient levels of exercise were unable to do this [45, 46, 54]. Indeed, even when barriers were not different between exercisers and non-exercisers, self-efficacy for exercise was higher in those who exercise regularly [32, 55, 56]. Moreover, self-efficacy for exercise mediated the association between pain and exercise: pain was no longer associated with exercise when self-efficacy was taken into account [51]. Finally, a quantitative study revealed that those RA patients who are more physically active also reported to have higher self-regulatory efficacy to overcome arthritis-related barriers to physical activity, while overall pain and number of flares were similar between patients with high levels of physical activity and those with low levels of physical activity [57]. Thus, the majority of the studies suggested that exercising patients might not be different from inactive patients in terms of their perceived barriers, but exercisers are able to manage or overcome these barriers more effectively than inactive patients.

### Perceived Facilitators for Physical Activity and Exercise

An overview of the facilitators for physical activity and exercise in patients with RA is shown in Table 2. In total, ten studies reported on facilitators, with the majority using qualitative methods [43–49, 58] and only two quantitative studies [39, 59]. Qualitative studies revealed that the most consistent RA-specific facilitating factor for regular physical activity and exercise was appropriate support from instructors and healthcare providers, which was reported in seven of nine studies [43–48, 58]. Similar findings were reported in the three studies that included only physician-diagnosed RA patients [43, 47, 48]. Social support or encouragement from family and friends (five of ten studies) also facilitated patients to participate in regular physical activity and exercise [43, 45, 46, 49, 58]. Indeed, those who currently exercise reported more support from family and friends than those who are inactive [36]. In addition, the experienced or expected positive physical effects (e.g. reducing stiffness and increasing strength) as well as the psychological effects (e.g. happiness) were important facilitators for regular physical activity [43] and exercise [44, 59]. It is important to note that the most frequently reported facilitating factors were consistently linked to barriers to regular physical activity and exercise. For example, reducing stiffness was a facilitator for physical activity and exercise, whereas experiencing stiffness was also mentioned as a barrier. Similarly, support from a healthcare provider was mentioned as a facilitator, whereas lack of this support was reported as a barrier. An exception is social support from significant others, which was only occasionally mentioned as a barrier [34]. Finally, it should also be acknowledged that social support was not a facilitating factor that is specific to patients with RA, but it is also often mentioned in other populations [60, 61].

### Perceived Benefits of Physical Activity and Exercise

A variety of RA-specific and generic benefits of participating in regular physical activity and exercise have been reported, as presented in Table 3. Of the 11 studies, five applied quantitative methods [25–27, 35, 39] and six used qualitative methods [24, 43, 45, 46, 58, 62]. Reported benefits did not differ between the quantitative and the qualitative studies. Physical activity and exercise were perceived to be an important contributor to symptom management, as mentioned in eight of ten studies [24, 27, 35, 43, 45, 46, 58, 62], such as pain relief [27] or distraction from pain [24], improvements in joint function [27, 35, 45, 46] and increased energy [45]. Together, these have a positive impact on daily tasks [35, 58]. These physiological benefits were also reported in studies only including patients with a physician diagnosis of RA [25, 27, 43, 62]. Feelings of independence and taking control were important perceived psychological benefits of physical activity and exercise [27, 35, 39, 45, 46, 58]. Similar to the experience of barriers, there did not seem to be a difference in perceived benefits between those who exercise and those who do not [53, 55], which is in line with the overall perception that RA patients are aware of the benefits of exercise in general and specifically for people with RA [23, 26, 48]. It is possible, though, that for inactive patients with RA, the perceived benefits are related to theoretical knowledge, whereas in those who are physically active the perceived benefits reflect the actual experience of such benefits. A recent study showed that, even though functional ability and social benefits of exercise were similar, those who regularly participated in exercise-related activities reported a broader range of physical and psychological benefits of regular exercise than those not regularly exercising [27].

### Barriers and Benefits Related to Physical Activity Behaviour

Even though barriers and benefits to physical activity and exercise in patients with RA are well-described, less is known about associations between physical activity behaviour and perceived barriers/benefits or the confidence to overcome these barriers (i.e. barrier efficacy). Perceived barriers were predictive of levels of physical activity or exercise in some [40, 41], but not in all, studies [53]. Care should be taken when interpreting and comparing these results, as different approaches have been used to quantify barriers in the literature. Whereas some studies evaluated barriers in terms of identification as well as the perceived impact of the barrier (i.e. how limiting is this barrier) [40, 41], others only measured the presence of a barrier [53]. Interestingly, levels of physical activity were associated with barriers when perceived impact as well as presence were taken into account [40, 41], but the presence of a barrier itself was not associated with exercise behaviour [53]. Therefore, it seems that barrier efficacy (i.e. the confidence to overcome a barrier) is a key aspect when exploring physical activity and the obstacles to regular engagement in physical activity. This is in agreement with the quantitative and qualitative studies comparing barriers between those who exercise regularly and those who do not reported above. The perceived barriers are similar between these groups of patients. Nevertheless, those who exercise have developed methods to overcome the indicated challenges. In other words, even though the barriers still exist in exercising patients, the impact of the barriers on physical activity and exercise behaviour is substantially reduced [32, 37, 45, 53, 54]. It should be acknowledged that these studies were not restricted only to RA patients; therefore, these findings need to be confirmed specifically in patients with a physician diagnosis of RA.

Perceptions of the benefits of physical activity have been shown to be positively related to participation in physical activity or exercise in most [34, 53, 63], but not all, cross-sectional studies [64]. In addition, lack of perceived benefits of regular physical activity was associated with physical inactivity [65] and, perhaps unsurprisingly, patients who complied with home exercise regimens reported more perceived benefits of exercise than those who did not comply with the exercise regimens [66]. However, it is worth noting that adherence to an exercise programme was not predicted on the basis of perceived exercise benefits prior to programme onset [55, 67] or self-reported physical activity post-intervention [68]. Given that the patients included in these studies were all about to start a physical activity or exercise intervention, they are likely to rate the benefits of exercise higher than the general RA population. However, this suggestion remains speculative, as a direct comparison between the perceived benefits in those about to start an exercise intervention and those of the general RA population is not possible due to the different methods used to quantify the benefits of exercise in existing studies. It should also be noted that different methods have been used to define and quantify physical activity and exercise (e.g. semi-structured interviews, questionnaires), which can influence the findings. For example, Greene and colleagues [64] made a distinction between leisure physical activity and household physical activity. Outcome expectations were associated with household physical activity; however, this association was not apparent for leisure physical activity or total physical activity [64]. In an observational longitudinal study, it was specifically leisure time physical activity and not work-related physical activity that was associated with improvements in functional ability in people with arthritis [69]. Therefore, the research to date suggests that modalities of physical activity are differentially related to (perceived) benefits of physical activity and perhaps exercise. This premise warrants further examination in patients with arthritis. As before, only a few studies have restricted their inclusion criteria to RA patients with a confirmed diagnosis; therefore, further studies are needed to explore these associations in this particular population.

Little is known about the interactive or additive effects of barriers or benefits in predicting physical activity or exercise. Multivariate path analyses revealed that only perceived benefits were associated with physical activity participation, with perceived barriers and health status not linked to exercise after controlling for potential modifying factors such as age, education, pain and disease duration [34]. Two further studies have explored the interactive effects of individual barriers in predicting exercise. Fatigue influenced the association between a combined measure of generic, non-arthritis-specific benefits and barriers with exercise participation. In the presence of high levels of fatigue, other barriers and benefits were not related to exercise, whereas when the levels of fatigue were low, generic barriers and benefits were associated with exercise [70]. Similarly, Der Ananian et al. [51] reported that when taking physical limitations into account, pain was no longer related to exercise levels, providing evidence for physical limitations as a mediating factor in the associations between exercise and pain. Thus, the existing evidence indicates that relationships between barriers and exercise behaviour are complex. Therefore, when examining predictors of exercise behaviour, the interaction between individual perceived barriers and/or benefits should be taken into account.

## Practical Implications and Recommendations

An overall summary of the findings related to the perceived barriers, benefits and facilitators is presented in Table 4. Patients with RA experience a range of disease-specific barriers to participating in regular physical activity and exercise, with fatigue and pain being the most commonly cited. Interestingly, there are similarities between the factors identified as barriers and benefits of physical activity and exercise in RA. It is worth reiterating that the main barriers are not different between those who exercise regularly and those who do not. In addition, only when a barrier was quantified in terms of its importance as well as its perceived impact on behaviour was it significantly predictive of physical activity [40, 41]. Therefore, the presence of barriers combined with the way the patient negotiates and effectively counters the barriers influences physical activity and exercise behaviour.

**Table 4:** Summary of findings related to rheumatoid arthritis-specific perceived barriers, perceived benefits and facilitators for physical activity and exercise in rheumatoid arthritis

Perceived barriers to physical activity and exercise	Perceived benefits of physical activity and exercise	Perceived facilitators of physical activity and exercise
Pain	Symptom management	Support
Fatigue	Pain relief and distraction	Exercise instructors
Mobility	Joint function	Health care provider
Stiffness	Independence	Family/friends
Lack of RA exercise programmes		Strength and aerobic capacity

*RA* rheumatoid arthritis

Given the influence of both the existence and the impact of barriers, both self-efficacy to overcome barriers and self-efficacy for exercise are important in this population. Even though different methods of assessment have been used to quantify self-efficacy, it is not surprising that those with higher levels of self-efficacy are more physically active [26, 53, 54, 57, 63–65, 71–73], have higher attendance at exercise programmes [32, 55, 56], and maintain physical activity post-intervention more frequently [68]. More specifically, self-efficacy to overcome arthritis-related barriers to physical activity was higher in those who participated in regular physical activity [57]. Further, self-efficacy for exercise can act as a mediator in the association between barriers or benefits and exercise. For example, the association between exercise and the perceived barrier of pain was no longer significant when self-efficacy for exercise was taken into account [51]. Only a few studies have assessed self-efficacy for exercise longitudinally. An association has been reported between changes in self-efficacy for exercise and changes in self-reported physical activity immediately following a 20-week intervention [73]. Interestingly though, self-efficacy was unchanged immediately after an exercise intervention, but lower 6–12 months after the programme than at pre-intervention baseline [74, 75]. Therefore, physical activity and exercise programmes should encourage the development of coping strategies to overcome the perceived barriers as well as enhancing self-efficacy for exercise, as these seem to be important predictors of adherence to exercise programmes and sustained physical activity and exercise behaviour.

In addition to emphasising the benefits of physical activity and exercise, education about exercise programmes for RA should be delivered to patients and healthcare providers [76, 77]. There is still uncertainty about what entails appropriate physical activity or exercise for patients with RA [24, 36, 48, 51], and patients do not feel that rheumatologists are able to give suitable advice on exercise [26, 37, 48], which was unfortunately confirmed by rheumatologists [24, 25, 36, 78]. A recent study revealed that almost all participating rheumatologists, clinical nurse specialists and physical therapists agreed that regular physical activity was an important goal for patients with RA [78]. However, there is a lack of confidence amongst healthcare providers about prescribing exercise and a lack of knowledge regarding referral programmes that are appropriate for RA [24, 25, 78, 79]. This is perhaps unsurprising given the lack of training for medical students related to the exercise sciences [80]. Therefore, appropriate and consistent health professional education about specific physical activity programmes, health communication and referral procedures appears warranted to get patients regularly involved in physical activity and exercise.

Given that social support from significant others is a facilitating factor for physical activity and exercise [36, 39, 43, 45, 46, 49], with discouragement or even disproval of exercise by significant others mentioned as barriers to physical activity [25, 36, 70], the impact of social support from close friends and relatives should be recognised. Therefore, educational materials about the benefits of exercise as well as suitable exercise programmes should be aimed towards the health professionals and patients as well as the relatives and friends of the patient. Given the differences between patients and healthcare providers in the perceptions of developing educational materials for patients [81, 82], it is important that all stakeholders are involved in the development of these educational materials.

It should be acknowledged that the studies reported in this review are not limited to patients with RA, with some studies including patients with other arthritic and inflammatory conditions. Given the limited availability of research centred only on RA patients with a confirmed diagnosis, it was decided that studies involving patients with a variety of arthritis diseases would be included as long as RA was specifically mentioned. To our knowledge, only one study conducted statistical sub-analyses to explore the impact of arthritis diagnosis. The diagnosis of RA versus osteoarthritis did not influence the association between outcome expectations and exercise time, but did impact on the association between self-efficacy for managing arthritis and total physical activity [64]. Nevertheless, the findings of the studies that included only RA patients are reported in each section. As can be seen from these reports, no substantial differences in reported barriers, facilitators and benefits for physical activity or exercise were found between the studies that included only RA patients and those that included a broader range of patients with arthritis (including self-diagnosed patients).

## Conclusion

RA not only affects the joints but can also influence general well-being and lead to an increased risk for CVD. Physical activity and exercise are effective methods to improve arthritis symptoms, enhance mental health and reduce the risk for CVD; however, the majority of patients with RA lead sedentary lifestyles. Nevertheless, patients with RA are aware of the health benefits of physical activity and the perception of the benefits is associated with physical activity behaviour. The literature points to several barriers to physical activity and exercise that are specific to the disease, such as pain and fatigue. Interestingly, the reported barriers do not differ between those RA patients who exercise regularly and those who do not. Exercising RA patients, however, appear more capable of overcoming these barriers. Therefore, there is a need for physical activity and exercise programmes customised for this population that support RA patients in overcoming barriers in order to sustain this important health behaviour. Given that encouragement from health professionals as well as friends and family were identified as important facilitators for physical activity, education about appropriate physical activity programmes and the benefits of physical activity programmes for RA should also target these significant others (see Table 5).

**Table 5:** Summary of findings and recommendations

Physically active patients are not different from inactive patients in terms of the perceived barriers, but those who are physically active are able to manage these perceived barriers more effectively than inactive patients Support from exercise instructors, healthcare providers and family/friends is an important facilitator for physical activity and exercise Perceived benefits are associated with physical activity, but knowledge about appropriate exercise programmes is lacking in patients and healthcare providers In order to increase the uptake and maintenance of physical activity behaviour and exercise, intervention programmes should
encourage the development of coping strategies to overcome the perceived barriers
increase the knowledge of the physical activity benefits for rheumatoid arthritis patients and healthcare providers
provide clear educational materials about appropriate exercise programmes aimed towards the healthcare professionals, the patients, and relatives and friends of the patient
